# Concurrent Radiation and Targeted Therapy for Papillary Craniopharyngioma: A Case Report

**DOI:** 10.7759/cureus.40190

**Published:** 2023-06-09

**Authors:** Sophia N Shah, Praneet C Kaki, Sohan S Shah, Sunjay A Shah

**Affiliations:** 1 Radiation Oncology, Christiana Care Health System, Newark, USA

**Keywords:** 3d conformal radiation therapy, concurrent chemo-radiation, papillary craniopharyngioma, braf v600e mutation, mek inhibitor

## Abstract

Craniopharyngiomas are rare epithelial malformations in the sellar or suprasellar regions of the craniopharyngeal ducts. Complete surgical resection is difficult due to the location of the base of the skull and the risk of injury to vital neurological structures. Fractionated radiation is effective in controlling residual tumors, but craniopharyngiomas can progress during treatment. The papillary subtype is driven by BRAF V600E mutations. Treatment with BRAF and MEK inhibitors alone has a response rate of 90% but a median progression-free survival of only 12 months. A 57-year-old female presented in May 2017 with complaints of headaches and blurriness in her right eye. Brain MRI demonstrated a 2 cm suprasellar mass engulfing the right optic nerve and optic chiasm. The patient underwent a transsphenoidal hypophysectomy with pathology consistent with a benign pituitary adenoma. Follow-up imaging in August, however, showed recurrence, and a re-resection was performed which surprisingly demonstrated papillary craniopharyngioma. Due to subtotal resection, the patient elected to proceed with intensity-modulated radiation therapy (IMRT) to the tumor bed in April of 2018 with an intended dose of 5400 cGy. After treatment with 2160 cGy in 12 fractions, the patient experienced visual deterioration and progression of the cystic tumor. The patient underwent another debulking procedure but due to rapid recurrence, an endoscopic transsphenoidal fenestration was performed. On postoperative imaging, a cystic mass was still engulfing the right optic nerve and chiasm. Due to the extended break and limited radiation tolerance of the optic chiasm, we elected to re-treat the tumor with an additional 3780 cGy IMRT in conjunction with one cycle of Taflinar and Mekinist, which was completed in August 2018. The cumulative dose to the optic chiasm was 5940 cGy.The patient had an excellent clinical response to treatment with the improvement of vision in her right eye. A brain MRI on 3/29/2019 demonstrated no residual craniopharyngioma. Four-year follow-on CT scan showed no evidence of tumor recurrence. The patient had preservation of vision and did not suffer any late neurological toxicity or new endocrine deficiency. Surgical resection and radiation were ineffective at treating our patient’s craniopharyngioma due to rapid cystic progression. This is the first case report in the literature detailing concurrent radiation therapy with BRAF and MEK inhibitors for papillary craniopharyngioma. Despite a suboptimal dose of radiation, our patient had no tumor recurrence and no late toxicity four years after treatment. This represents a potentially novel treatment strategy in this challenging entity.

## Introduction

Craniopharyngioma is a rare epithelial malformation of the sellar or suprasellar regions arising from the craniopharyngeal ducts. Craniopharyngiomas have an incidence of 0.5-2.0 cases per million population per year [1]. They can be classified as adamantinomatous or squamous papillary, depending upon their morphology and genesis [2]. Found primarily in children, adamantinomatous craniopharyngiomas derive from the epithelial cells of the craniopharyngeal ducts and produce non-enhancing hyperintense cysts. Squamous papillary craniopharyngiomas are mainly present in middle-aged adults and arise from the metaplasia of the cell rests found in the buccal mucosa and produce hypointense cysts [3]. 

Despite being Grade 1 neoplasms, they are considered malignant due to their critical location and high recurrence rate. The average three-year survival rate has been reported as 87.6% in a study based on a large US population-based database between 2004-2008 [4]. Due to their location, craniopharyngiomas can present with hypothalamic and pituitary deficiencies along with visual impairment and headaches [5]. The initial treatment choice depends upon the tumor's proximity to vital structures. If the tumor is localized and distant from the pituitary or optical tracts, complete resection is favored. However, if the tumor involves critical neurovascular structures, partial surgical resection and/or cystic drainage followed by postoperative fractionated radiation is often utilized to minimize normal tissue complications [5]. Long-term tumor control is similar for either treatment strategy. Craniopharyngiomas can recur despite treatment, with 10-year and 15-year recurrence rates of 33% and 40%, respectively [6].

The papillary subtype is driven by V600E point mutations in the BRAF oncogene, which is involved in the RAS/MAPK signaling pathway. These mutations are detectable in 96% of papillary craniopharyngiomas. Treatment with BRAF and MEK inhibitors alone has a response rate of 90% but median progression-free survival of only 12 months [7]. Neoadjuvant use of these inhibitors prior to radiation therapy has been reported and is an evolving strategy. To the best of our knowledge, this is the first report of BRAF and MEK inhibitors used concurrently with radiation for craniopharyngiomas. 

## Case presentation

**Figure 1:**
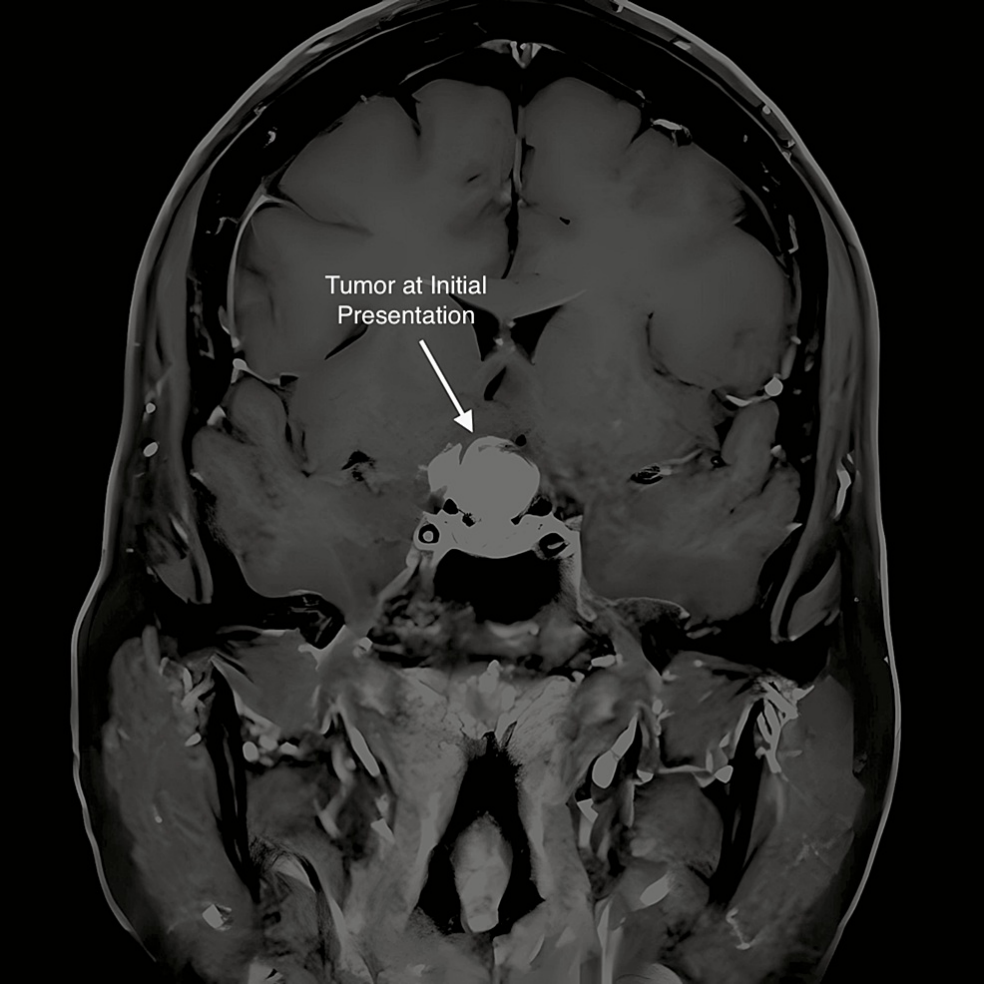
Preoperative mid-coronal MRI with contrast; this shows a 1.8 cm x 1.6 cm x 1.9 cm suprasellar craniopharyngioma with a large cystic component resulting in mass effect on the right prechiasmatic optic nerves and optic chiasm

A 57-year-old female presented in May 2017 with complaints of a six-month history of progressive headaches, nausea, and loss of vision in her right eye. On examination, she complained of 8/10 right-sided headaches and was found to have a bitemporal hemianopsia, right greater than left. Finger-to-nose alternating movements were impaired on the left. Complete blood count and complete metabolic profile were normal. An MRI scan (Figure 1) revealed an enhancing suprasellar mass measuring 1.8 cm x 1.6 cm x 1.9 cm with effect upon the right prechiasmatic optic nerves and optic chiasm associated with a cystic component. The patient underwent a transsphenoidal resection but with no improvement in her symptoms. The pathology report at that time suggested pituitary adenoma. Postoperative imaging noted a substantial residual mass with a persistent anterior cystic component causing mass effect. Pituitary function tests obtained on 6/16/17 were normal except for a mildly elevated prolactin level of 48.2 (normal 4.8-23.3).

**Figure 2:**
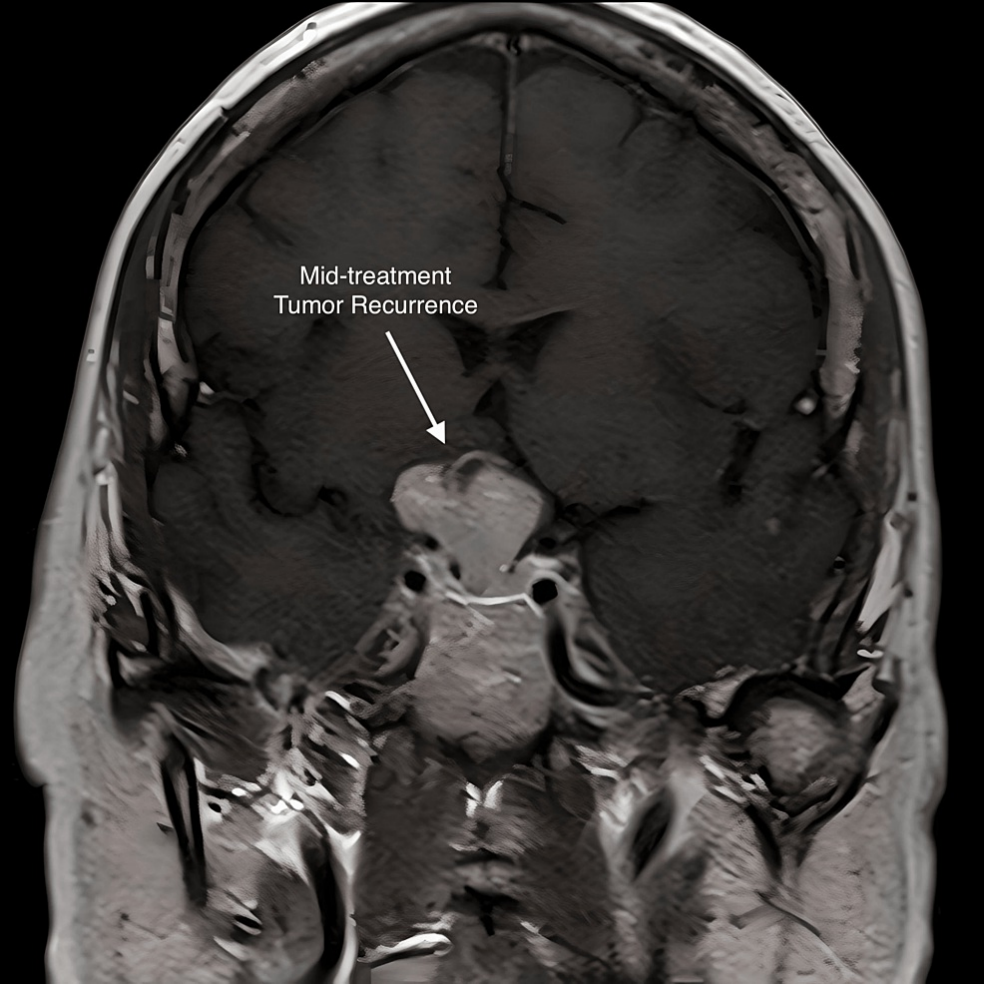
Post-operative mid-coronal MRI with contrast; this shows a 2.6 cm x 2.4 cm x 2 cm craniopharyngioma with substantial interval progression with a persistent anterior cystic component causing mass effect

**Figure 3:**
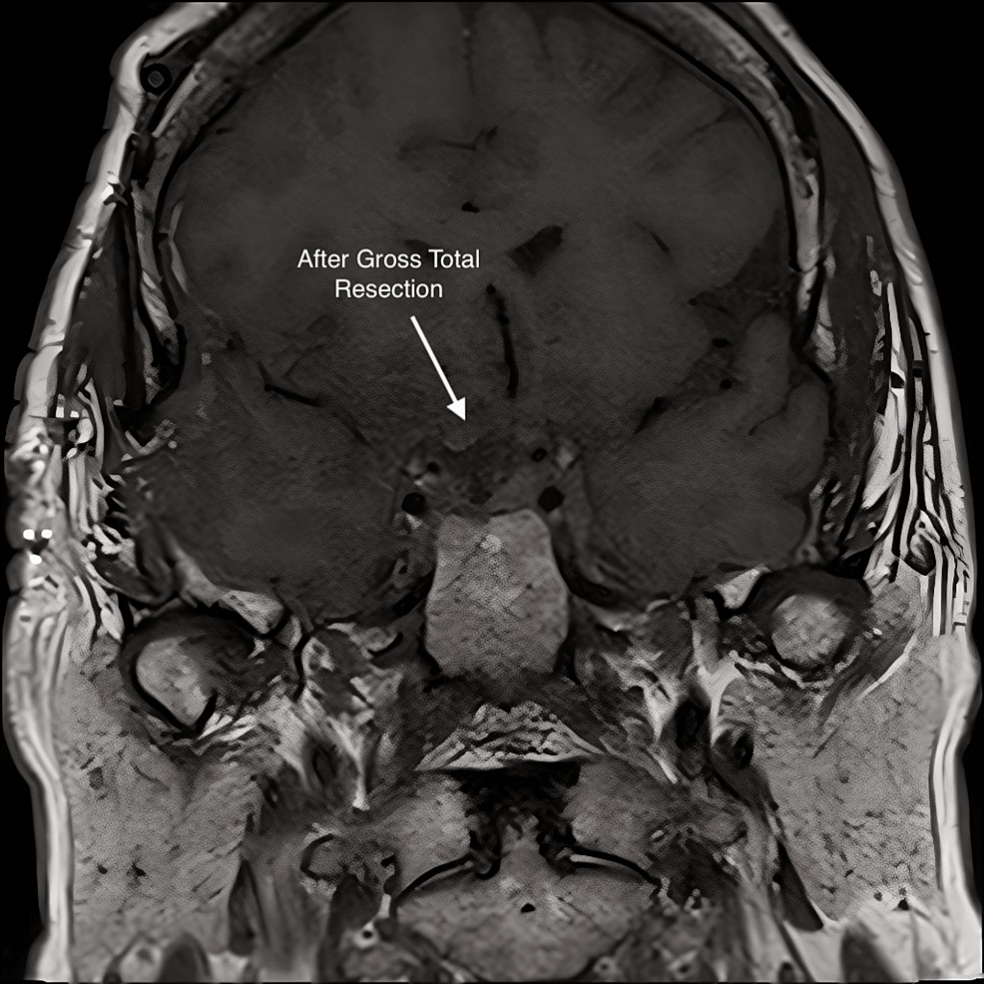
Mid-coronal MRI with contrast; this was taken after subfrontal craniotomy with gross total resection resulting in resolution of headaches and “brighter” vision

**Figure 4:**
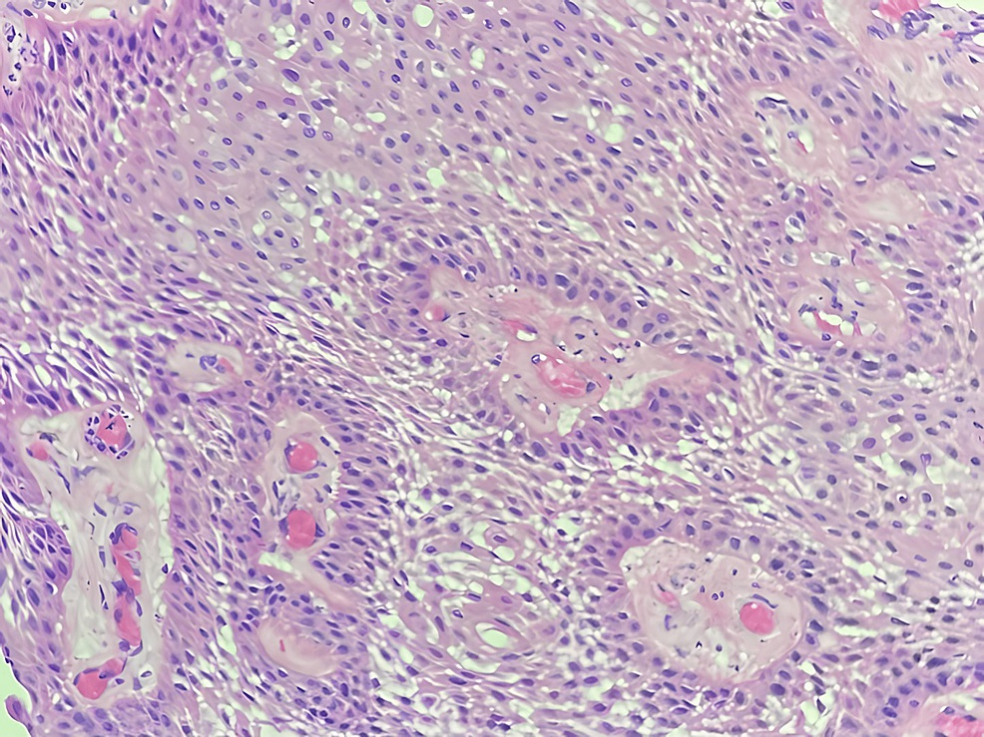
Pathology showed non-keratinized squamous epithelium and polymorphonuclear cell infiltration consistent with a papillary craniopharyngioma

Follow-up imaging in August 2017 demonstrated mass growth to 2.6 cm x 2.4 cm x 2 cm (Figure 2). The patient underwent a subfrontal craniotomy with gross total resection as shown in Figure 3, after which the patient reported resolution of headaches and “brighter” vision. The pathology (Figure 4), surprisingly, was papillary craniopharyngioma, as evidenced by a non-keratinized squamous epithelium and polymorphonuclear cell infiltration. The tumor was positive for the BRAF V600E mutation, the hallmark of papillary craniopharyngioma. The previous biopsy was reviewed, and it was established that there was no definitive evidence of an adenoma. The patient had a good postoperative recovery and was prescribed pituitary hormone replacement therapy. 

**Figure 5:**
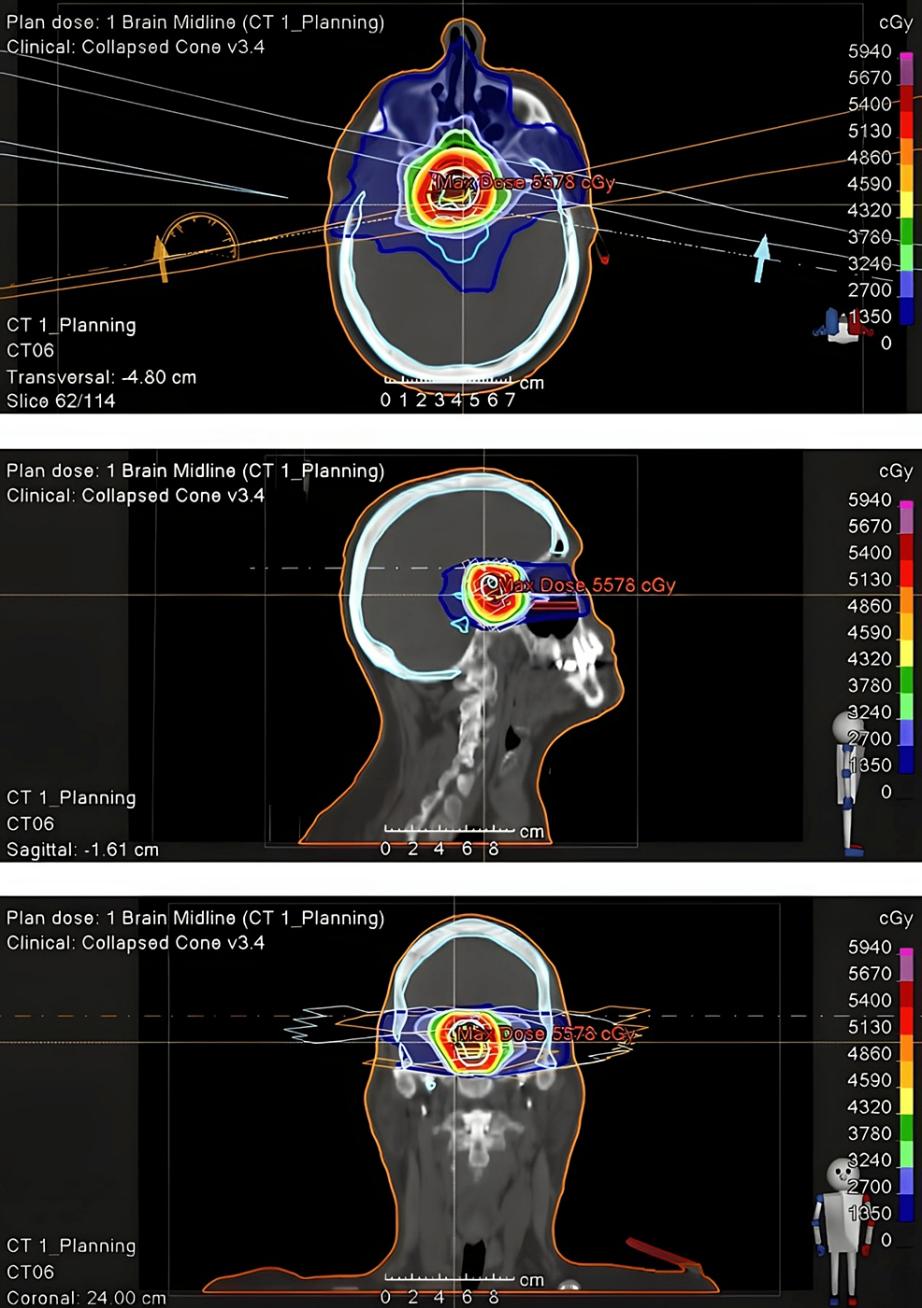
Intensity-modulated radiation therapy radiation plan prescribed to a dose of 5400 cGy in 30 fractions

**Figure 6:**
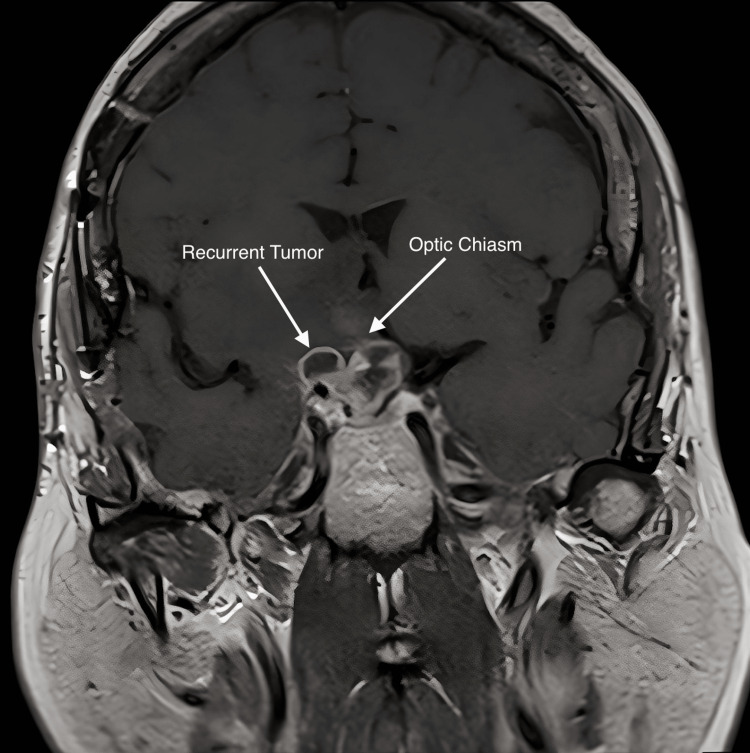
Mid-coronal MRI with contrast; this shows clinical progression and cystic enlargement of the tumor after 2160 cGy in 12 fractions

**Figure 7:**
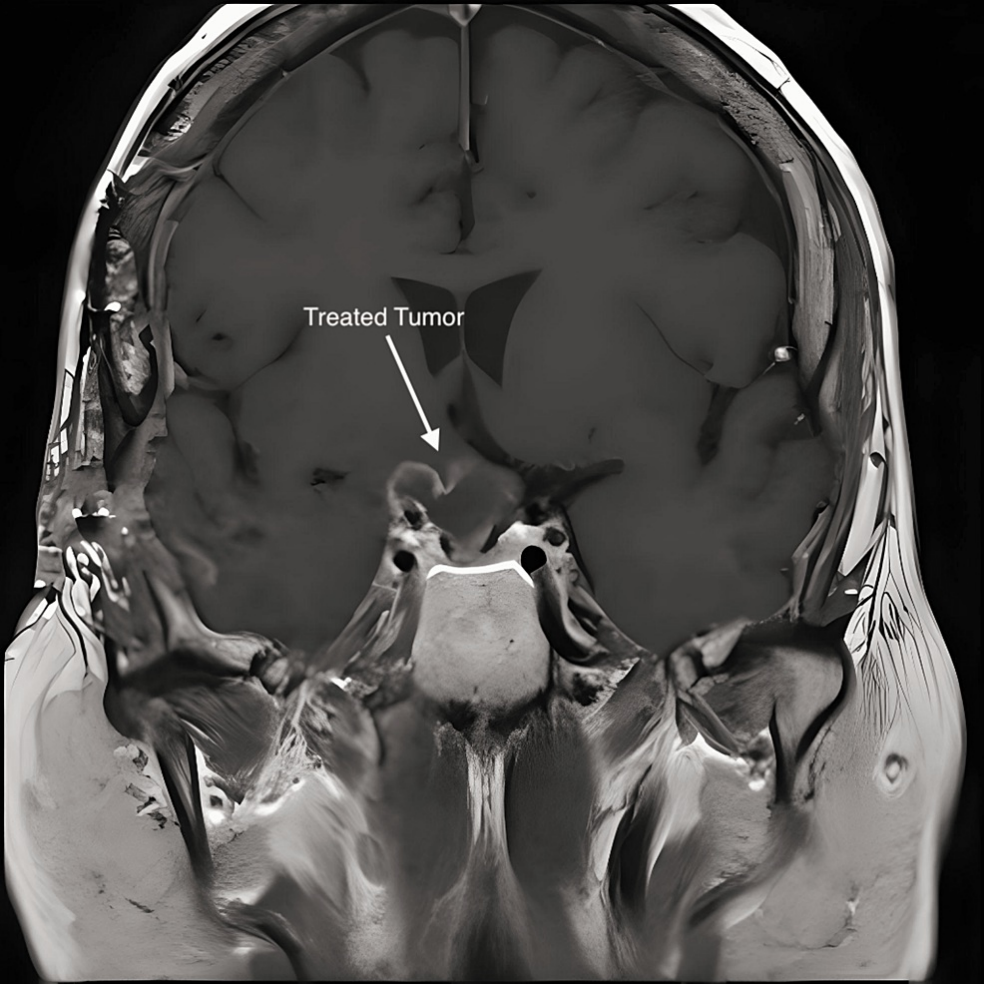
Mid-coronal MRI with contrast; taken after the debulking procedure, the scan demonstrates persistent cystic compression of the chiasm and the right optic nerve

The patient started standard postoperative intensity-modulated radiation therapy (IMRT) with an intended total dose of 5400 cGy in 30 fractions (Figure 5), starting on 4/5/2018. Unfortunately, after receiving 2160 cGy in 12 fractions, she developed complete right eye blindness with headaches and cystic enlargement of the tumor on MRI (Figure 6). Following a subsequent debulking procedure on 4/24/2018, the patient felt some relief in her symptoms. However, she had a rapid recurrence of blindness, and MRI (Figure 7) demonstrated cystic compression of the chiasm and the right optic nerve on 5/31/18. The patient underwent an endoscopic transnasal transsphenoidal fenestration of the mass with placement of a drain on 6/7/18. She subsequently reported improvement of vision on the left side but minimally on the right side. Post operative MRI demonstrated a decrease of the cystic mass, but the residual was still engulfing the right optic nerve and chiasm, and she was functionally blind in the right eye. 

Despite the four-month interruption of treatment, we elected to continue her course of IMRT, and the patient received another 3780 cGy for a total of 5940 cGy, which was completed on 8/3/18. Due to the V600E BRAF mutation, we elected to treat the patient concurrently with one cycle of dabrafenib and trametinib. During the latter part of the treatment, the patient became very fatigued and dehydrated. She was given 4 mg of Decadron twice a day to relieve her brain edema with a good response and then given a gradual Decadron taper. She also developed excoriations of her right arm. By the end of treatment, she had improved vision in the nasal aspect of her right visual field. Subsequent to treatment, the patient was hospitalized and required surgery for diverticulitis. She also required treatment for a pelvic abscess and suffered a pulmonary embolism. She refused any further chemotherapy. 

**Figure 8:**
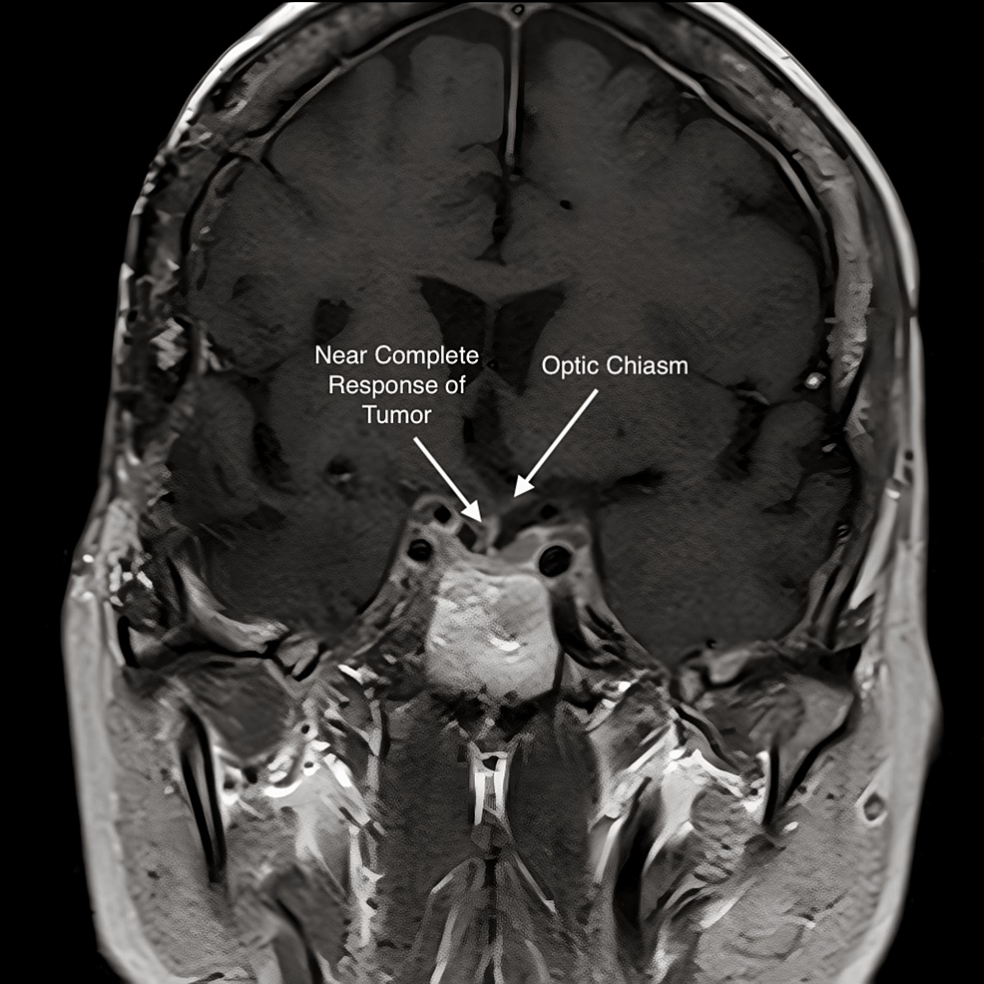
Mid-coronal MRI with contrast; the seven-month post-treatment scan shows a near-complete response to treatment

MRI brain scan (Figure 8) taken on 3/29/2019 demonstrated resolution of the craniopharyngioma, At the follow-up on 4/8/2019, the patient reported improved symptoms without headaches or other neurologic complaints. The patient was not compliant with follow-up. A repeat brain CT scan at four years demonstrated no evidence of disease recurrence. The patient had preservation of residual vision with no new endocrinopathies.

## Discussion

Craniopharyngiomas are rare benign tumors that generally have a favorable prognosis if located distant from vital neurological structures such as the hypothalamic-pituitary system and optic tracts and are amenable to complete surgical resection. However, when involving these critical structures, the standard approach is a partial resection followed by adjuvant radiation therapy in order to reduce the high risk of recurrence [5]. Despite the original goal of administering 5400 cGy in 30 fractions, the IMRT in this case had to be discontinued at 2160 cGy (12 fractions) due to rapid cystic growth causing visual loss. Multiple debulking procedures followed by endoscopic defenestration and cystic drainage were necessary to arrest the tumor growth, resulting in a four-month interruption of the radiation treatment. The optic chiasm is one of the most sensitive structures in the body with an accepted standard dose constraint of 5400 cGy in the literature [8] to minimize the risk of radiation-induced blindness. We thought that we could discount some of the 2160 cGy already administered in the first treatment due to the four-month break and we elected to administer an additional 3780 cGy resulting in a total accumulated optic chiasm dose of 5940 cGy. A dose of 3780 cGy is considered to be insufficient to control gross craniopharyngioma [9], and we were faced with the dilemma of whether the potential benefit was worth the toxicity of treatment. In order to maximize the chance for long-term local control, the patient consented to be treated with concurrent radiation and BRAF/MEK inhibitors. 

BRAF and MEK inhibitors have primarily been developed and studied for the treatment of melanomas with BRAF V600E mutations. Dual treatment with both BRAF and MEK inhibitors is used because of the compounded effect of MEK inhibitors, which block the downstream effects of the mitogen-activated protein kinase involved in the RAF-MEK-ERK (MAPK) pathway. The benefits of dual treatment were suggested by a phase III clinical trial for melanoma, in which the median progression-free survival was 9.4 months in the BRAF/MEK group versus 5.8 months in the BRAF-only group [10]. 

A review article by Chowdhary et al. describes the potential synergistic benefits of treating melanoma brain metastases (MBM) with a combination of BRAF inhibitors and radiation, as evidenced by several retrospective studies. This could possibly be a result of a radiosensitization effect in melanomas cell lines, as they become more prone to radiation therapy following treatment with BRAF inhibitors [11]. In fact, a case report about the success of using BRAF inhibitors along with radiation to treat melanoma suggested that radiation is able to increase the permeability of the blood-brain barrier to improve the effectiveness of the inhibitors [12]. However, there are also several potential complications that could arise from this dual-therapeutic method, such as radiodermatitis, radionecrosis, and intracranial hemorrhage. There are currently several prospective clinical trials underway that could shed more light on the safety and efficacy of combined BRAF inhibitors and radiation treatment for MBM. In addition, decreasing the tumor size by first treating it with BRAF inhibitors would reduce the amount of radiation required, leading to less toxicity [12,13].

With the realization that papillary craniopharyngiomas are driven by BRAF V600E mutations, several case reports of dramatic clinical and radiographic responses to BRAF and MEK inhibitors have been published. BRAF and MEK inhibitors alone have a response rate of 90% but the median progression-free survival has been only 12 months [14]. In a study from the Washington University School of Medicine, the neoadjuvant use of BRAF/MEK inhibitors was used to treat papillary craniopharyngioma to initially reduce the tumor size, resulting in less radiation administered to reduce toxicity [15]. This suggests that BRAF and MEK inhibitors are not very effective as a single modality, but may be potent when used in conjunction with radiation. We decided to proceed with concurrent BRAF/MEK inhibitors and radiation in this case because of the potential synergistic effect with the suboptimal radiation dose allowed by the limited radiation tolerance of the optic chiasm.

BRAF/MEK inhibitors are associated with significant vascular and dermatologic toxicities [16]. In our case, only one cycle of dabrafenib and trametinib was administered concurrently with radiation. The patient suffered grade 2 acute CNS toxicity during treatment including marked fatigue and headaches which responded to steroids. She also had grade 2 excoriations of the arm consistent with the known dermatologic toxicity of BRAF/MEK agents. After treatment, the patient required surgery for diverticulitis complicated by pelvic abscess and pulmonary embolus likely consistent with a grade three vascular toxicity which may have also been related to the BRAF/MEK inhibitors. No further targeted therapy was given. Despite the acute toxicity, there were no late neurovascular effects to the optic apparatus or pituitary gland and the patient had long-term preservation of vision. 

## Conclusions

In this case, we have demonstrated that concurrent BRAF and MEK inhibitors with suboptimal radiation therapy doses can result in long-term control of papillary craniopharyngioma. This suggests a synergistic effect between these modalities since either therapy alone was unlikely to result in long-term disease control. There is currently an ongoing clinical trial testing the efficacy of using BRAF and MEK inhibitors to treat papillary craniopharyngioma; the pending results could potentially provide further validation of this novel treatment methodology.
